# Impact of Mutational Status on Intracellular Effects of Cell‐Permeable CaaX Peptides in Pancreatic Cancer Cells

**DOI:** 10.1002/cbic.202401076

**Published:** 2025-04-24

**Authors:** Merlin Klußmann, Martin Matijass, Ines Neundorf

**Affiliations:** ^1^ Department of Chemistry and Biochemistry Institute of Biochemistry University of Cologne Zuelpicher Str. 47a 50674 Cologne Germany

**Keywords:** cancer, (cell‐penetrating) peptides, posttranslational modification, prenylation, Ras proteins

## Abstract

Prenyltransferases add a lipid group to the cysteine of a CaaX motif of proteins. This posttranslational modification enables proteins to attach to membranes where they are essential hubs for signaling, trafficking, and apoptosis. Recently, cell‐permeable CaaX‐peptides are developed as possible tools to interfere with the prenylation machinery. These peptides cause cytotoxic effects, particularly in KRas mutant pancreatic cancer cells (PANC‐1) in which they also alter downstream signaling of Ras proteins. Herein, the aim is to get more clues about the relevance of the mutational status of KRas. Therefore, the activity of CaaX‐peptides in KRas wildtype BxPC‐3 and KRas mutated PANC‐1 cells is compared. CaaX‐peptides differently influence these two cell lines, although they internalize pretty much to the same extent. Indeed, an altered KRas plasma membrane localization in PANC‐1 cells is observed, probably induced by disturbed KRas prenylation based on the presence of CaaX‐peptides. The impact of CaaX‐peptides on KRas signaling is likely dependent on the KRas mutation in PANC‐1 cells in which they further trigger effects on KRas‐dependent regulators, e.g., Neurofibromin −1 (NF1) and son of sevenless homolog 1 (SOS1). All in all, CaaX peptides are identified as promising tools for studying and manipulating the function of therapeutically important prenylated proteins.

## Graphical abstract

This work deals with intracellular effects of cell‐permeable CaaX‐peptides, designed to influence prenylated proteins like KRas. The results show that after CaaX‐1 treatment, not only KRas expression and signaling are specifically altered in mutated pancreatic cancer cells, but also plasma membrane localization of KRas is affected. Thus, CaaX‐peptides are promising tools to manipulate prenylation‐dependent proteins.

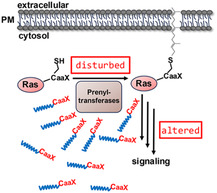

## 1 Introduction

Protein prenylation is a posttranslational modification present in all eukaryotes, in which either a farnesyl group (C_15_) or a geranylgeranyl group (C_20_) is irreversibly attached to the cysteine of a *C*‐terminal CaaX motif.^[^ ^1^, ^2^, ^3^ ^]^ This thioether linkage is catalyzed by farnesyltransferase (FTase) or geranylgeranyltransferase type 1 (GGTase I), respectively, which are soluble enzymes within the cytosol.^[^ ^4^, ^5^, ^6^, ^7^ ^]^ After cleavage of the *C*‐terminal tripeptide ‘aaX’ by Ras Converting CAAX Endopeptidase 1 (RCE‐1)^[^ ^8^, ^9^ ^]^ and *C*‐terminal methylation by Isoprenylcysteine Carboxyl Methyltransferase (ICMT),^[^ ^10^, ^11^ ^]^ the prenylated proteins are transported to membranes, particularly the membrane of the Golgi apparatus, the ER, and the plasma membrane (PM), to enable their specific localization and activity by membrane anchoring.^[^ ^8^, ^11^, ^12^, ^13^, ^14^, ^15^ ^]^ The most studied members of prenylated proteins comprise the small GTPases Ras, from which two isoforms (H‐Ras, N‐Ras) and two splice variants (K‐Ras‐4 A and K‐Ras‐4B) exist.^[^ ^7^, ^16^, ^17^ ^]^ Often located at the PM, these proteins act as molecular switches in key signaling pathways regulating cell proliferation, differentiation, and apoptosis.^[^ ^7^, ^17^ ^]^ However, due to their important role, mutations in Ras proteins usually lead to dysfunctions, causing severe diseases. Indeed, about 20%–30% of all cancer and even 90% of all pancreatic cancer types show gain‐of‐function mutations in Ras proteins.^[^ ^7^, ^18^, ^19^, ^20^, ^21^, ^22^ ^]^

Treatment of Ras‐mediated cancers proved challenging as Ras proteins are poorly addressable by small molecules.^[^ ^23^, ^24^ ^]^ Recently, there has been progress in this field as several KRas G12C specific inhibitors have been approved by the Food and Drug Administration (FDA), or are in clinical studies including Adagrasib (MRTX‐849, Mirati),^[^ ^25^, ^26^ ^]^ Sotorasib (AMG‐510, Amgen),^[^ ^27^, ^28^ ^]^ and JNJ‐74 699 157 (ARS‐3248, J&J).^[^ ^29^, ^30^, ^31^ ^]^ These inhibitors bind covalently to the mutated cysteine of this KRas variant and force conformational changes of the switch regions resulting in increased affinity to guanosine diphosphate (GDP) over guanosine triphosphate (GTP) and tumor growth inhibition.^[^ ^29^, ^32^ ^]^ Despite this progress, treatment of KRas mutants remains a major challenge, as statistics from the COSMIC database show that only ≈12% of all KRas‐mediated cancers have a KRas G12C mutation.^[^ ^33^ ^]^ However, these KRas G12C inhibitors might serve as a template, and indeed, inhibitors targeting KRas G12S,^[^ ^33^, ^34^ ^]^ G12R,^[^ ^33^, ^35^ ^]^ and G12D mutants^[^ ^33^, ^36^ ^]^ are currently in development.

In addition to the direct targeting of Ras proteins indirect ways have also come into focus that affect upstream regulators, downstream effectors, membrane association, or posttranslational modifications. In this context, farnesyltransferase inhibitors have been developed to inhibit farnesylation of Ras, thereby preventing its association with the PM. Although the results were highly promising for HRas, the outcome for NRas and KRas was disappointing. While HRas is exclusively prenylated by FTase, NRas and KRas can undergo cross‐prenylation by GGTase I that rescues the lipidation of Ras and thus its activity.^[^ ^33^, ^37^, ^38^, ^39^, ^40^, ^41^, ^42^ ^]^

To address this issue and to better understand the prenylation process, we recently designed chimeric cell‐permeable CaaX‐peptides (CaaX‐1‐3) containing the cell‐penetrating peptide (CPPs)C18* and sequences derived from Ras proteins.^[^ ^43^ ^]^ These peptides accumulated to a high extent in various cell lines, dependent on the presence of an intact CaaX‐motif as well as on the accessibility of isoprenoids. For instance, several control peptides having no CaaX motif, a SaaX motif in which Cys was substituted for Ser, or also scrambled variants, were all significantly less or even not taken up by cells. Moreover, inhibition of the mevalonate pathway by statins leads to a decreased cellular uptake of CaaX‐peptides. Also, in silico interaction of CaaX‐1 (GLRKRLRKFRNKSKTKCVIM‐OH) with FTase was studied by our group in recent work.^[^ ^44^ ^]^ Here, we found out that CaaX‐1 has a high affinity for FTase and even outcompetes shorter control peptides. However, in our preliminary work, we recognized specific cytotoxicity toward cancer cells, particularly in KRas‐mutated pancreatic cancer cell lines. Notably, CaaX‐1 was particularly able to alter Ras‐related signaling pathways in KRas‐mutant pancreatic cancer cells (PANC‐1).

In this work, we set out to test the influence of CaaX‐peptides on KRas mutated and wildtype cells to get a deeper understanding of the involved mutational status of KRas. For this, we chose the pancreatic cancerous cell lines PANC‐1 and BxPC‐3, respectively, and performed comparative studies in cytotoxicity and internalization activity. Moreover, we examined if CaaX‐peptides, particularly CaaX‐1, would differentially influence Ras localization and Ras signaling pathways in both tested cell lines.

## 2 Results and Discussion

### 2.1 Cytotoxicity and Cellular Uptake Profiles of CaaX‐Peptides

Our previous findings revealed significant cytotoxic activities of CaaX peptides (namely CaaX‐1, CaaX‐2 and CaaX‐3; see Table S1, Supporting Information) when they came in contact with several different cancer cell lines, including KRas G12D mutated PANC‐1 cells.^[^ ^43^ ^]^ Pancreatic ductal adenocarcinoma (PDAC) is one of the most lethal malignancies with a very poor outcome. Nearly 90% of all PDACs are KRas mutated, and the G12D mutation is the most prevalent.^[^ ^18^ ^]^ Therefore, it is of high interest to identify substances that specifically affect KRas signaling cascades. Finding a specific biological effect of our CaaX‐peptides may potentially lead to new agents in this direction. However, in our former study, we did not involve pancreatic KRas wildtype cells (e.g., BxPC3) as a further control. The BxPC3 cell line complements the PANC‐1 experimentally, and differences between both cell lines would provide insights into the biological function of the peptides and if their activity is related to the KRas mutation. Our hypothesis was that CaaX‐1 peptides would differentially affect both cell lines due to the different genetic abnormalities of KRas and, thus, the activation state of KRas. We also included HFF‐1 cells in our studies, which represent a healthy control cell line. In addition, we tested serine control peptides, namely SaaX‐1 and SaaX‐2, that were not prenylated.^[^ ^43^ ^]^

**Figure 1:**
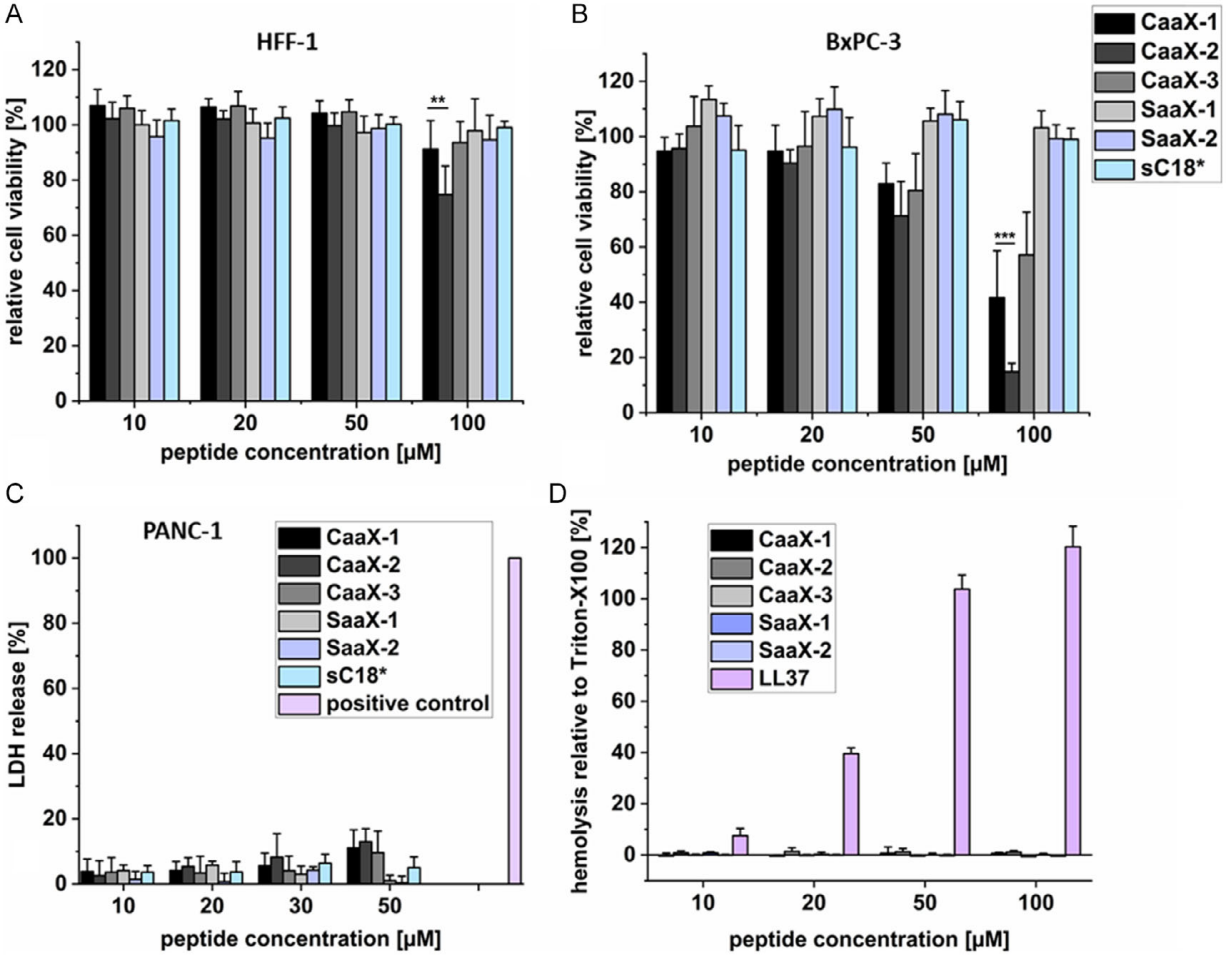
Cytotoxicity profiles of CaaX‐peptides in A) HFF‐1 and B) BxPC‐3 cells after 24 h of peptide treatment. Viability of untreated cells was set to 100%; significances were determined by student's *t*‐test. (*P < 0.05, **P < 0.005, ***P < 0.0005). C) Effects of CaaX‐peptides on the membrane integrity of PANC‐1 cells. Cells treated with lysis solution (100% lactate dehydrogenase (LDH) release) was used as positive control. D) Hemolytic activity of peptides analyzed in human red blood cells. Cells treated with 10% Triton‐X100 served as a positive control and was set to 100% hemolytic activity. Assays were performed in triplicate and *n* = 3 in A,B,D) or *n* = 2 in C).

First, we incubated the cells for 24 h with CaaX‐peptides but no cytotoxic effects in HFF‐1 cells were detected for CaaX‐1 and CaaX‐3, while CaaX‐2 caused some minor effects at 100 μm peptide concentration (**Figure** **1**A). We observed increasing cytotoxic effects in BxPC‐3 cells starting at peptide concentrations of 50 μm (Figure 1B). In fact, when treating BxPC‐3 cells with 100 μm peptide concentration, cell viability was dramatically reduced (to ≈40% for CaaX‐1, ≈15% for CaaX‐2 and ≈60% for CaaX‐3, respectively). Compared to our previous results, the cytotoxic effects of all tested peptides on BxPC3 cells were considerably lower compared to the recently tested PANC‐1 cells. Here, cells were dramatically affected at 50 μm and at 100 μm, almost all cells were dead.^[^ ^43^ ^]^ Interestingly, this effect was somehow more pronounced to CaaX‐1 as it was less toxic in BxPC‐3 and HFF‐1 cells than CaaX‐2 but similarly toxic to CaaX‐2 in PANC‐1 cells, which could potentially be related to its KRas origin. Moreover, these finding likely hints to a more specific cytotoxic activity in KRas‐mutated cells since both BxPC3 as well as HFF‐1 are KRas wildtype cells.

To exclude that the observed higher cytotoxicity in PANC‐1 cells was related to membrane‐disturbing effects of the peptides, we performed a membrane integrity assay and analyzed the cells after 30 min of peptide treatment. No or only minor LDH release was detected when cells were treated with a peptide concentration of up to 50 μm (Figure 1C), concluding that the high cytotoxicity of the CaaX‐peptides is not due to membrane disruption but more likely caused by other intracellular effects.

Next, we tested the hemolytic activity of CaaX‐peptides using human red blood cells to see if membrane perturbation would also be neglectable for this cell model. Thus, erythrocytes were incubated with the peptides for 24 h and after centrifugation, the supernatant was screened for heme groups indicating hemolytic activity. The results revealed no such influence of the CaaX‐peptides and their controls up to 100 μm concentration, indicating a high tolerance to the peptides (Figure 1D). In contrast, the control peptide LL37, which is known to be highly membrane‐active,^[^ ^45^ ^]^ showed high hemolytic effects since already at 50 μm, almost all red blood cells were disrupted.

**Figure 2:**
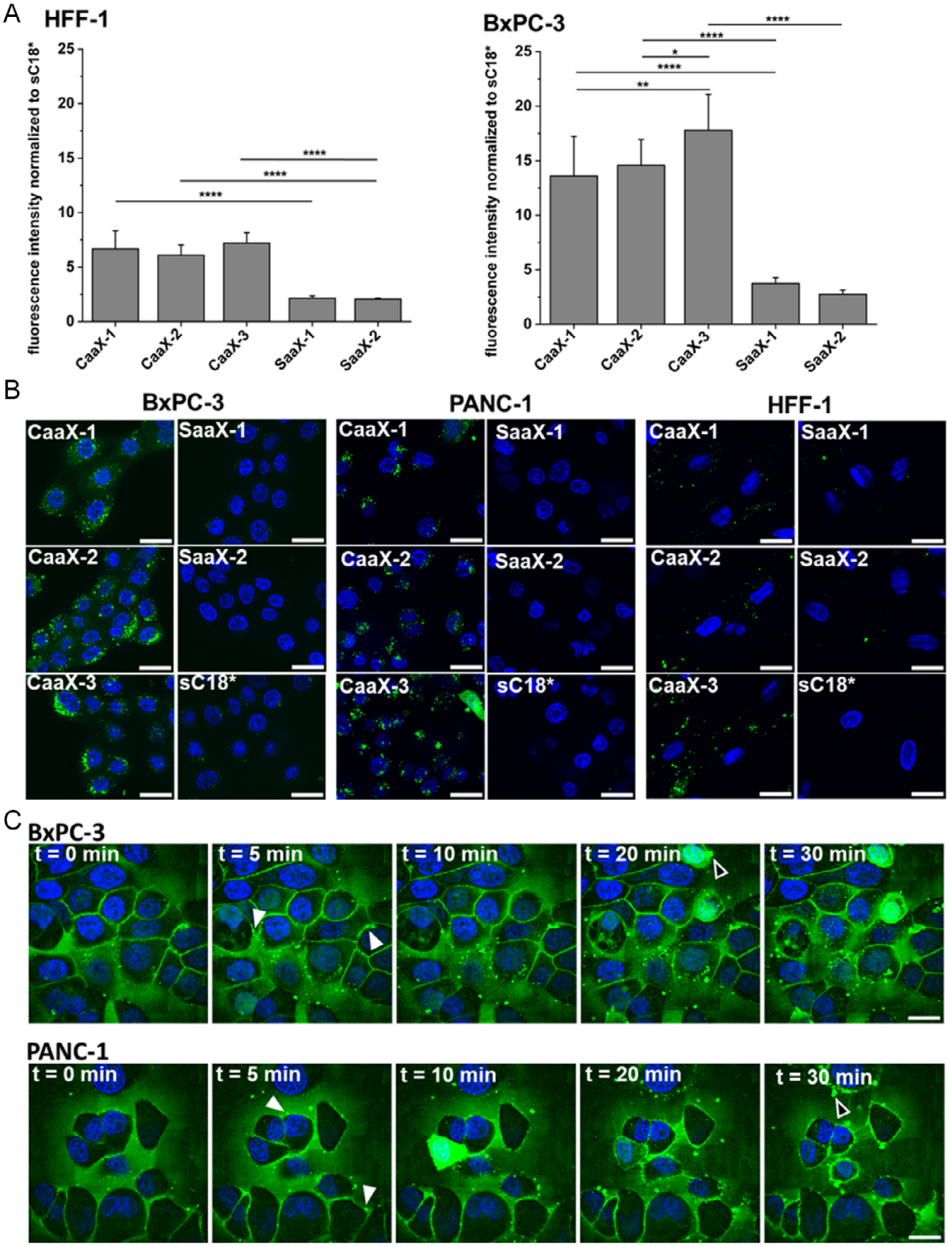
A) Cellular uptake of 10 μm CF‐labeled CaaX‐peptides in HFF‐1 and BxPC‐3 cells after 30 min measured by flow cytometry. The fluorescence intensity of CF‐sC18* were set to 1. (*n* = 3) B) Microscopic analysis of 10 μm of the CF‐labeled peptides in HFF‐1 and BxPC‐3 cells after 30 min. Blue: nuclear Hoechst 33 342 stain, green: CF‐labeled peptide; scale bars are 20 μm. (*n* = 2). C) Time lapse imaging of 10 μm CF‐CaaX‐1 using BxPC‐3 and PANC‐1 cells over 30 min period. Scale bars are 10 μm. Images were processed with Fiji. White filled arrows indicate peptide aggregations at the PM potentially referring to nucleation zones or liquid droplets. Black filled arrows: Cells releasing fluorescent vesicles presumably containing CF‐labeled CaaX‐1. Movies are included in the SI.

Then, we asked if cytotoxicity would be related to the cellular uptake of CaaX‐peptides. Thus, we performed flow cytometry analysis in BxPC‐3 and HFF‐1 cells, showing that carboxyfluorescein (CF)‐labeled CaaX‐peptides indeed translocated to a lesser extent in HFF‐1 cells, while the uptake was higher in BxPC3 cells (**Figure** **2**A). This was in agreement to our former results, where we also measured a higher uptake in cancerous PANC‐1 cells compared to non‐cancerous cells.^[^ ^43^ ^]^ We assumed that this observation is due to a different PM composition of cancerous cells compared to healthy cells. Indeed, cancerous cells are characterized by a more negatively charged outer PM supporting interaction with cationic cell‐penetrating peptides.^[^ ^46^, ^47^, ^48^, ^49^ ^]^ In addition, the cellular uptake of CaaX‐peptides was obviously higher than that of the SaaX control peptides in all tested cell lines, emphasizing the relevance of a functional CaaX motif.^[^ ^43^ ^]^

We also performed fluorescence microscopy in the three cell lines to get a better impression of the intracellular distribution of CaaX‐peptides. The results supported the findings of the flow cytometry measurement showing higher uptake of CaaX‐peptides in BxPC‐3 and PANC‐1 cells compared to HFF‐1 cells (Figure 2B). Again, internalization of CaaX‐peptides was increased compared to the SaaX control peptides. Furthermore, a mainly punctate distribution pattern of the peptides within the cells was visible in the three cell lines, suggesting a vesicular uptake or probably aggregation of the peptides. However, there was also a diffuse distribution of green fluorescence visible, particularly in BxPC‐3 cells indicating a possible direct uptake and cytosolic spread of the internalized CaaX‐peptides.

### 2.2 Intracellular Fate of CaaX‐1

CaaX‐1 includes parts of the secondary signal of KRas4B, therefore, we assumed a more selective intracellular effect of CaaX‐1 on KRas‐mutated cells compared to the other two peptides. Therefore, we performed the next experiments only with CaaX‐1 to investigate in more detail the intracellular fate when in the presence of either BxPC‐3 or PANC‐1 cells. First, we analyzed if the cellular uptake of CaaX‐1 would be time‐dependent and observed that CaaX‐1 seemed to translocate somewhat faster in BxPC‐3 cells compared to PANC‐1 cells (Figure 2C and movies 1‐2). Furthermore, CaaX‐1 treatment led to high peptide accumulation at the plasma membrane. Interestingly, this peptide accumulation increased over time in PANC‐1 cells, assuming that peptides might also be present at the cytosolic site of the lipid bilayer. Additionally, aggregate‐like structures were visible at the plasma membrane, particularly in PANC‐1 cells, which we assigned to so‐called nucleation zones. Recently, the relevance of nucleation zones for the cellular uptake of arginine‐rich CPPs was postulated.^[^ ^50^ ^]^ Additionally, peptides have been shown to form liquid microdroplets through temperature or pH‐dependent phase separation, involving interactions between hydration water and specific amino acid residues.^[^ ^51^, ^52^ ^]^ We cannot exclude that such microdroplets were also potentially present when the cells were treated with CaaX‐1. Indeed, such aggregate‐like structures have recently been identified when cells were incubated with a mixture of the lytic peptide L17E trimer [FcB(L17E)_3_] and Alexa488‐IgG, and might also play a role in CaaX‐1 cellular uptake.^[^ ^52^ ^]^ Furthermore, both cell lines also displayed a few cells that showed an extremely intense and diffuse green signal of CaaX‐1, suggesting a very high cytosolic location. Also, many cells released peptide‐containing vesicular structures, representing potentially extracellular vesicles. This was again more frequently observed for PANC‐1 cells.

**Figure 3:**
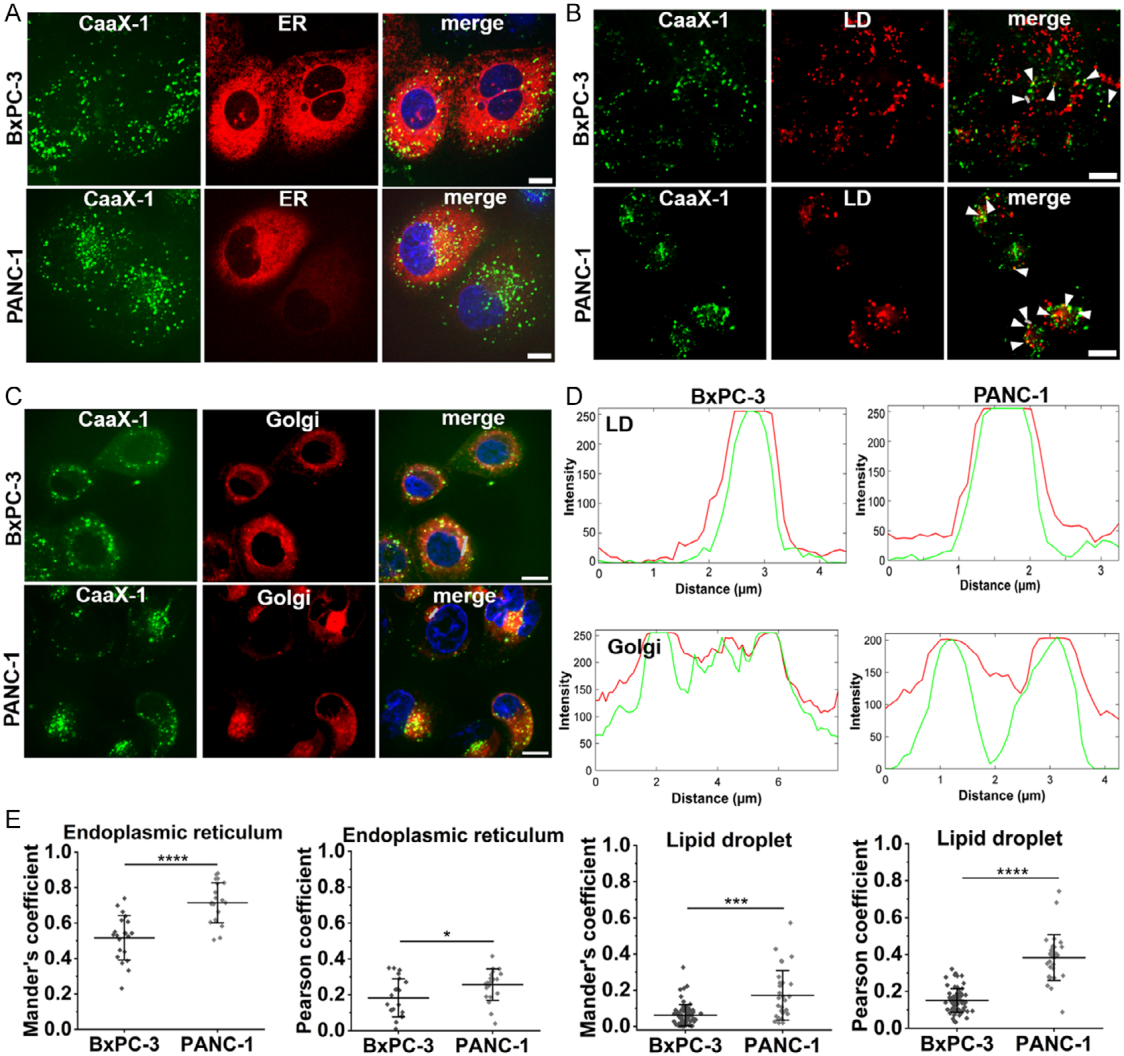
A) Colocalization studies of CaaX‐1 with the ER in BxPC‐3 and PANC‐1 cells after cells were treated with 10 μm of CF‐labeled CaaX‐1 for 30 min. (*n* = 2) B) Colocalization studies of CaaX‐1 with lipid droplets in BxPC‐3 and PANC‐1 cells after 30 min incubation with 10 μm of CF‐labeled CaaX‐1. Colocalization is indicated by white arrows. (*n* = 2) C) Colocalization studies of CaaX‐1 with the Golgi apparatus after treating BxPC‐3 and PANC‐1 with 10 μm of CF‐CaaX‐1 for 30 min. (*n* = 2) Blue: nuclear Hoechst 33 342 stain, green: CF‐labeled CaaX‐1, red: the respective organelle stain. Scale bars are 10 μm. D) Line profiles of regions of interest (gray lines) of the green and red channels of the merged images of B,C). E) Pearson correlation and Mander's overlap coefficients from the merged images from A,B) determined with Fiji. Significances were determined by student's *t*‐test. (*P < 0.05, **P < 0.005, ***P < 0.0005, ****P < 0.00005).

We were then interested to know which subcellular compartments would be relevant for intracellular CaaX‐1 peptide accumulation and used several markers for co‐staining experiments. To study colocalization with the ER, PANC‐1 or BxPC‐3 cells were transfected with an red fluorescent protein (ER‐RFP) fusion protein that stains the ER with red fluorescence, since a live stain did not lead to conclusive results. After 48 h, cells were treated with 10 μm CF‐labeled CaaX‐1 for 30 min and imaged by confocal fluorescence microscopy. In all samples, yellow spots were visible, indicating that CaaX‐1 colocalized with the ER in both cell lines tested (**Figure** **3**A). We also quantified this overlapping fluorescence by using two different methods, namely Pearson's correlation and the determination of Mander's overlap coefficients (Figure 3E). Normally, Pearson's correlation coefficient ranges from −1 to 1, where −1 stands for no colocalization, 0 for random colocalization, and 1 for perfect colocalization. We determined Pearson's correlation coefficients of 0.18 ± 0.10 in BxPC‐3 cells and 0.26 ± 0.09 in PANC‐1 cells, assuming that colocalization with the ER is more relevant for CaaX‐1 peptides in PANC‐1 cells. This hypothesis was corroborated after calculating Mander's overlap coefficient, too. The values typically range between 0 to 1, where 0 indicates no overlap and 1 corresponds to perfect overlap. Here, we measured 0.52 ± 0.12 for BxPC‐3 and 0.71 ± 0.11 for PANC‐1 cells, again showing higher values when CaaX‐1 peptides were taken up in PANC‐1 cells. Overall, the detected colocalization might hint at a further peptide modification at the membrane of the ER, for instance by RCE‐1 and ICMT.

Since we also observed aggregate‐like structures, we were interested if these could be allocated to peptide accumulation within lipid droplets, which are highly hydrophobic lipid‐rich cellular organelles. CaaX‐1 peptides are possibly equipped with a farnesyl anchor after internalization and are then able to interact with such lipid bodies. Indeed, when incubating both cell lines for 30 min with 10 μm of CF‐labeled CaaX‐1 and a lipid droplet stain,^[^ ^53^ ^]^ several spots appeared that suggested peptides present at lipid droplets (Figure 3B). Notably, line profiles of regions of interest demonstrated an almost perfect overlay of the green (peptide) and red (lipid droplets) signals (Figure 3D). As already mentioned, potential prenylation of CaaX‐1 by prenyltransferases, such as FTase, would increase the lipophilicity of the peptide and enable its association to lipid droplets. Interestingly, the determined Pearson (BxPC‐3: 0.15 ± 0.06; PANC‐1: 0.38 ± 0.12) and Mander's (BxPC‐3: 0.06 ± 0.06; PANC‐1: 0.17 ± 0.0.13) coefficients were both higher in PANC‐1 cells (Figure 3E). However, only a subset of the CF‐labeled CaaX‐1 peptides seemed to colocalize with lipid droplets suggesting a more unspecific binding of the peptide to these compartments.

Lastly, we studied colocalization of CaaX‐1 with the Golgi apparatus since the yellow spots around the nucleus observed in Figure 2B might indicate localization of CaaX‐1 in this organelle. Noteworthy, the phenotype of the Golgi apparatus of BxPC‐3 and PANC‐1 differed, as the Golgi stain was more punctate and distributed around the nucleus in BxPC‐3 cells, while it was more concentrated at one side of the nucleus in PANC‐1 cells (Figure 3C). However, this phenomenon was also recently described by others.^[^ ^54^, ^55^ ^]^ Indeed, colocalization of CaaX‐1 with the Golgi apparatus was observed in both cell lines and proven by yellow spots in the merged images. Again, line profiles of regions of interest in both cell lines supported this observation (Figure 3D). Maybe this partial enrichment of CaaX‐1 was caused by two different effects such as its prenylation as well as its high positive charge, as the Golgi apparatus has one of the most negatively charged membranes.^[^ ^56^ ^]^ This might have led to golgi membrane association of CaaX‐1 by hydrophobic interactions via the prenyl tail and electrostatic interaction between the peptide and the phospholipid head groups.

### 2.3 Intracellular Effects of CaaX‐1 on KRas Signaling

**Figure 4:**
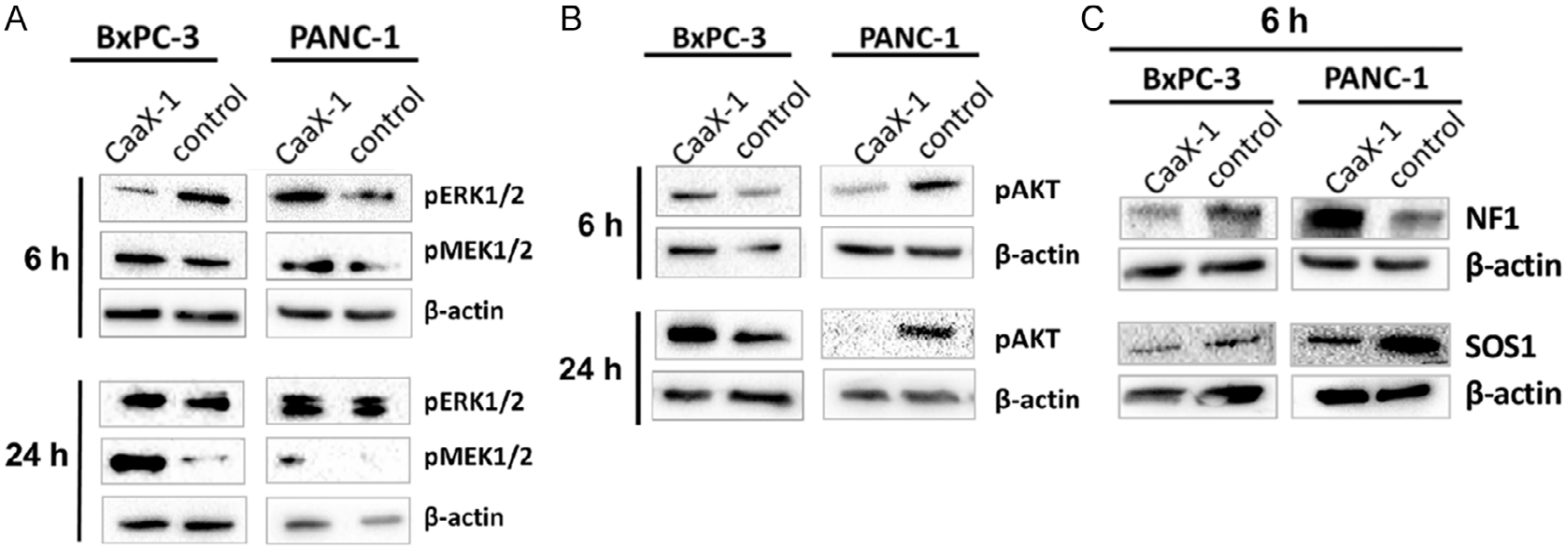
Influence of CaaX‐1 on the expression and phosphorylation levels of members of the A) RAF‐MEK1/2‐ERK1/2 and B) PI3K‐AKT‐mTOR pathways after treating BxPC‐3 and PANC‐1 cells for 6 h (left) and 24 h with the peptide. C) Influence of the CaaX‐1 peptide on the expression levels of NF1 and SOS1 after treatment of BxPC‐3 and PANC‐1 cells with 30 μm of CaaX‐1 peptide for 24 h. (*n* = 3) A) and C) The same loading control is shown for pERK1/2, pMEK1/2 and NF1 (PANC‐1, 6 h incubation) since the results were obtained from the same cell lysate samples.

Our recent studies suggested that CaaX‐1 is able to influence downstream signaling cascades of Ras proteins in PANC‐1 cells, including members of the extracellular signal‐regulated kinase (ERK)/mitogen‐activated protein kinase (MAPK)‐ and phosphatidylinositol 3‐kinase (PI3K)/protein kinase B (AKT)‐pathways.^[^ ^43^ ^]^ Indeed, there is an increasing body of evidence that AKT is amplified in pancreatic cancer, contributing significantly to tumor cell growth and proliferation.^[^ ^57^ ^]^ Moreover, this effect is more pronounced in cells with KRas mutation compared to wildtype KRas. Therefore, we were eager to know if our recently obtained results would also be consistent with these findings and tested the influence of CaaX‐1 of the phosphorylation state in PANC‐1 and BxPC‐3 cells. Indeed, we observed higher expression levels of AKT/pAKT in PANC‐1 compared to BxPC‐3 cells (**Figure** **4**B). More of interest was that treatment with CaaX‐1 peptides lead to a switch to the Raf‐MEK1/2‐ERK1/2 pathway in PANC‐1 cells (Figure 4A). Thereby, CaaX‐1 seemed to act relatively fast since the expression level of pAKT was reduced already after 6 h. The change to the MEK/ERK pathway was somehow complete after 24 h and agreed to our former studies.^[^ ^43^ ^]^ This finding is still interesting since substances inhibiting the PI3K/AKT pathway may have potential as novel treatments against adenocarcinoma of the pancreas. On the other side, the influence of CaaX‐1 on BxPC‐3 cells was presumably more unspecific. It has already been shown that the PI3K/AKT pathway is only moderately activated in BxPC3 cells compared to the MEK1/2‐ERK1/2 pathway, although both pathways are normally constitutively active.^[^ ^57^, ^58^ ^]^ Also, in our studies, we observed a moderate activation of the PI3K/AKT cascade after 24 h only. However, after 24 h both pathways seemed to be activated. Collectively, the results presented in Figure 4A,B conclude a more specific activity of CaaX‐1 toward KRas‐mutated cells.

In the next panel of experiments, we examined how other Ras‐dependent regulators were altered by CaaX‐1. For instance, the tumor suppressor Neurofibromin‐1 (NF1) functions as a general negative regulator of Ras oncoproteins.^[^ ^59^ ^]^ It belongs to the family of GTPase‐activating proteins that trigger the hydrolysis of Ras‐bound GTP to GDP, which in turn leads to an inactive state of Ras. It has been noted that in NF1‐deficient tumors, Ras‐signaling is constitutively activated.^[^ ^60^ ^]^ Moreover, for KRas G12D mutated PDAC, it has been demonstrated that NF1 inactivation can substitute for oncogenic KRas^[^ ^61^ ^]^ and facilitate PDAC progression. Therefore, we tested the CaaX‐1 influence on NF1 expression after 6 h incubation using either BxPC‐3 and PANC‐1 cells (Figure 4C). We found that NF1 is highly abundant in BxPC‐3 cells but is less expressed after CaaX‐1 treatment, pointing to activation of the PI3K/AKT pathway in BxPC‐3, what we already demonstrated in the former experiment (Figure 4B). In addition, our observations were consistent with the recent finding that knockout of NF1 in PDAC activates AKT signaling.^[^ ^61^ ^]^ More of interest was that in PANC‐1 cells, which are known to be NF1 inactivated, CaaX‐1 treatment led to high expression of NF1. This corroborated our results of a less significant role of AKT‐signaling for these experimental conditions (Figure 4B) and implied that an increased expression level of NF1 decreases activation of AKT in KRas G12D mutant cells. Recent studies also showed that NF1 inhibits wildtype KRas activity in heterozygous KRas mutant cells,^[^ ^62^ ^]^ which is also true for PANC‐1 cells and could further explain our results.^[^ ^63^ ^]^

Besides NF1, we identified SOS1 as an interesting candidate for our studies, a positive Ras regulator that stimulates the exchange of Ras‐bound GDP to GTP.^[^ ^64^ ^]^ It was recently shown that the genetic ablation of SOS1 in a mouse model of KRas G12D‐driven lung adenocarcinoma induced tumor shrinking and regression.^[^ ^65^ ^]^ Moreover, intravenous tail injection of SOS1‐less, KRas G12D tumor cells into wildtype mice, or of KRas G12D tumor cells into SOS1^KO^/KRAS^WT^ mice, further underscored the importance of SOS1 for the tumor progression of KRas G12D‐driven lung adenocarcinoma.^[^ ^65^ ^]^ To see if similar effects would also count for CaaX‐1 treatments, we investigated SOS1 expression levels in PANC‐1 and BxPC‐3 cells. Strikingly, when cells were inspected after 6 h of CaaX‐1 addition, the level of SOS1 was considerably decreased in KRas‐mutated PANC‐1 cells, while there was no significant effect on the SOS1 level in BxPC‐3 cells. This would again be consistent with the observed reduction in AKT activation by CaaX‐1, as SOS1 downregulation would lead to reduced KRas activity. Moreover, less activated KRas would further diminish SOS1 activity as only activated Ras has the ability to allosterically stimulate SOS1.^[^ ^66^ ^]^

### 2.4 Analysis of KRas Expression and Localization

**Figure 5:**
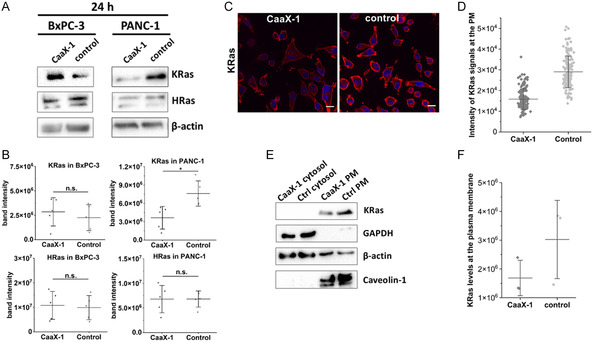
Effects toward KRas expression and localization after CaaX‐1 treatment. A) Influence on the expression level of HRas and KRas in BxPC‐3 and PANC‐1 cells after 24 h treatment with CaaX‐1. (*n* = 5) B) Band intensity of KRas (top row) and HRas (bottom row) upon CaaX‐1 treatment relative to the control. C) Immunostaining of KRas in CaaX‐1 treated and untreated cells. Blue: nuclear Hoechst 33 342 stain, red: anti‐KRas‐Alexa 555 conjugate. Scale bar: 20 μm. (*n* = 3) D) Quantification of KRas signal intensity at the PM in CaaX‐1 treated and control cells. One spot represents the intensity of KRas at the PM of one cell. Error bars correspond to the standard deviation. E) KRas levels at the PM and the cytosol in CaaX‐1‐treated (3 h) and untreated control cells. (*n* = 3) F) Quantification of the blot intensities of E). Error bars represent standard deviations. Significance was determined by student *t*‐test (*P < 0.05, **P < 0.005, ***P < 0.0005).

Based on the findings thus far, we wondered if CaaX‐1 would also directly impact KRas expression and localization in PANC‐1 and BxPC‐3 cells. Notably, Western blot studies revealed that KRas levels were significantly decreased after treating PANC‐1 cells for 24 h with CaaX‐1, while in BxPC‐3 cells KRas expression seemed to be unaffected or even somewhat increased. From this we assumed again a more specific impact of CaaX‐1 in the KRas mutant cell line (**Figure** **5**A,B). Interestingly, HRas levels were unaffected by CaaX‐1 in both BxPC‐3 and PANC‐1 cells. This was somehow in line with the influence of CaaX‐1 on the NF1 levels, as Ramakrishnan et al. have shown that *NF1* deletion increased the levels of KRas‐GTP, but did not increase the levels of HRas‐GTP or NRas‐GTP.^[^ ^59^ ^]^ Furthermore, this likely supports a selective effect of CaaX‐1 toward KRas.

**Figure 6:**
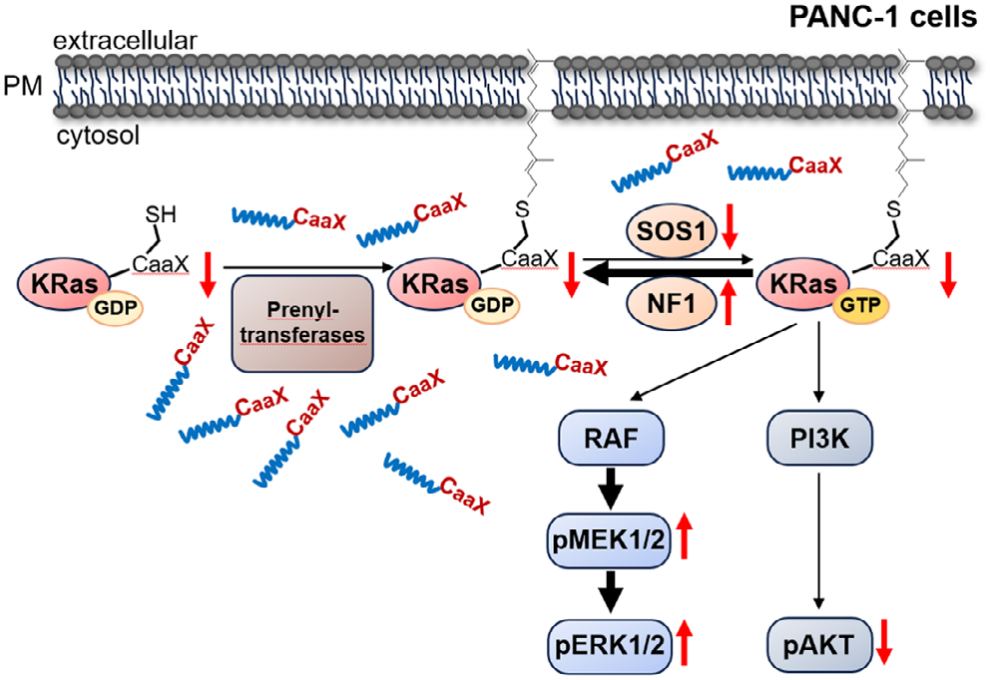
Possible influence of CaaX‐1 on KRas‐mutated PANC‐1 cells. CaaX‐peptides interfere with the intracellular posttranslational machinery of S‐prenylation, e.g., the prenyltransferases. Thereby, they will then probably be prenylated, which we did not show here for the sake of clarity. However, they likely alter KRas membrane localization to the PM. Also, the interaction with further regulators of KRas, such as NF1 and SOS1, is affected, leading to disturbed downstream signaling.

We also investigated whether CaaX‐1 would influence KRas localization to the PM in PANC‐1 cells, as Ras proteins require PM localization in order to activate signaling cascades and to promote oncogenic cell proliferation. After 3 h CaaX‐1 treatment, a reduced intensity of the KRas signal at the PM was present (Figure 5A,B). This presumably explains the observed effects on downstream signaling pathways, e.g., the PI3K‐AKT pathway, in PANC‐1 (Figure 4B). We further supported the findings by Western blot analysis. In fact, the fractions of membrane and cytosol of PANC‐1 cells revealed decreased KRas levels at the PM while there was no signal detected for KRas within the cytosol (Figure 5C,D), probably due to degradation processes of KRas. Taken together, the results underscore that CaaX‐1 not only specifically affects downstream signaling or regulators of KRas in KRas mutated PANC‐1 cells but also has a direct effect on intracellular KRas expression and localization (**Figure** **6**).

## 3 Conclusion

Previously, we developed cell‐permeable CaaX‐peptides comprising a cell‐penetrating peptide and the C‐terminal region of Ras proteins, including a CaaX motif and further amino acids of the secondary signal. We demonstrated that these peptides had substantial effects on cytotoxicity and Ras signaling of KRas‐mutated PANC‐1 cells.

Within this present work, we aimed to investigate whether these effects were related to the mutational status of KRas and therefore compared KRas wild type (BxPC‐3) and G12D mutated PANC‐1. While the cellular uptake into the two cell lines was more or less similar, cytotoxicity was obviously higher in the KRas‐mutated cells, and this cytotoxicity was presumed to be the result of other intracellular events. Colocalization studies revealed partial localization of the CaaX‐1 peptide to the Golgi, the ER, and lipid droplets, and that these latter two organelles are more relevant for the localization of CaaX‐1 in PANC‐1 cells. By receiving a potential prenylation modification, the hydrophobicity of the peptide is considerably increased, which allows association to these organelle membranes. However, the phenotype was somehow similar for the two tested cell lines, so we investigated in more detail how CaaX‐1 affected KRas.

We found that CaaX‐1 has a different impact on KRas signaling and KRas regulators in the two pancreatic cancer cell lines tested, suggesting specificity with respect to the KRas‐mutated PANC‐1 cells. Moreover, immunofluorescence and Western blot studies highlighted that both the PM localization and the overall expression levels of KRas in PANC‐1 cells appeared to be impaired by CaaX‐1, most likely by affecting the prenylation process or the transport to the PM. This is particularly interesting and might pave the way to the development of alternative new therapeutic peptides for KRas‐mutated cancer types.

In the future, we will further investigate how exactly CaaX‐peptides influence KRas signaling cascades and which mechanisms are behind it. In this context, we will also focus on the interaction between CaaX‐peptides and prenyltransferases, in particular FTase.

All in all, we are convinced that these peptides display interesting tools to study and likely manipulate proteins, other than KRas, that are dependent on posttranslational S‐prenylation.

## 4 Experimental Section

4.1

4.1.1

##### Peptide Synthesis and Purification

All studied peptides were synthesized by solid phase peptide synthesis (SPPS) according to the Fmoc/tBu strategy with an automated Syro I peptide synthesizer (MultiSynTech). Sequences are depicted in Table S1, Supporting Information. Preloaded Wang resins or Rink amide resin were used as solid support. CaaX‐ and SaaX‐peptides were synthesized as previously described by us.^[^ ^43^ ^]^ For cellular uptake and intracellular localization studies, peptides were *N*‐terminally labeled on the resin with 5(6)‐carboxyfluorescein (CF). For this, 5 equivalents (eq.) each of CF, Oxyma, and N,N’‐diisopropylcarbodiimide (DIC) were incubated with the resin at room temperature overnight. Peptide cleavage from the resin was performed by trifluoroacetic acid (TFA)/thioanisol/1,2‐ethanedithiol (90/7/3, v/v/v) treatment at room temperature for 3 h. Ice‐cold diethyl ether was used for peptide precipitation. Lyophilized peptides were purified by preparative reversed phase (RP)‐high pressure liquid chromatography (HPLC) (Elite LaChrom, Hitachi, Chiyoda, Japan) on a VP 250/16 Nucleodur 100‐5 C18ec column (Macherey‐Nagel, Düren, Germany). Analytical data were obtained by RP‐HPLC (Hewlett Packard Series 1100, Agilent; column: EC 125/4.6 NUCLEODUR 100‐5 C18ec, Macherey‐Nagel, Düren, Germany) using a linear gradient of 10%–60% Acetonitrile in water with 0.1% formic acid for 15 min and subsequent electrospray‐ionization mass spectrometry analysis (LTQ‐XL, Thermo Scientific (Waltham, Massachusetts, USA)). UV‐spectra were generated by analytical RP‐HPLC using a linear gradient of 10%–60% ACN in water with 0.1% TFA in 15 min.

##### Cell Culture

Cells were cultured as subconfluent monolayers in petri dishes (BxPC‐3 and PANC‐1) or T‐75 flasks (HFF‐1) at 37 °C and 5% CO_2_ with humidified atmosphere. BxPC‐3 and PANC‐1 cells were cultured in RPMI 1640 with 10% FBS and 4 mm L‐glutamine. HFF‐1 cells were grown in Dulbecco's Modified Eagle's Medium (high glucose level) supplemented with 15% Fetal Bovine Serum (FBS) and 4 mm L‐glutamine. Cells were split when they reached 70%–80% subconfluency by detaching cells with 0.5 mg ml^−1^ trypsin‐ethylenediaminetetraacetic acid (EDTA).

##### Cell Viability Assay

Cells were seeded in a 96‐well plate (HFF‐1: 1.6 × 10^4^ cells, BxPC‐3: 2.2 × 10^4^ cells) and grown to about 90% subconfluency. Cells were incubated with various peptide concentrations (100, 50, 20, and 10 μm) in a serum‐free medium at 37 °C for 24 h. On the next day, cells were washed with phosphate‐buffered saline (PBS) and incubated with a 10% resazurin solution in serum‐free medium at 37 °C for 1–2 h. Cells treated with 70% EtOH for 10 min served as positive control and untreated cells at negative control. The plate then was measured at 595 nm after excitation at 550 nm using a Tecan infinite M200 plate reader.

##### Membrane Integrity Assay

2.3 × 10^4^ PANC‐1 cells were seeded in a 96‐well plate and grown to 80%–90% subconfluency. On the next day, cells were incubated with different peptide concentrations in serum‐free medium at 37 °C for 30 min followed by equilibration to room temperature for 20 min. Cells treated with lysis solution from the kit (CytoTox‐ONE^TM^ Homogeneous Membrane Integrity Assay, Promega), which contains Triton‐X100, served as positive control and untreated cells as negative control. After adding a volume CytoTox‐ONE reagent (contains resazurin) equal to the volume of medium present in the well, the plate is gently shaken for 30 s and subsequently incubated at 22 °C for 10 min. Finally, a stop solution was added, and the emission at 595 nm was measured after excitation at 550 nm using a Tecan infinite M200 plate reader.

##### Hemolysis Assay

Purified human red blood cells (SER‐10MLRBC, Zenbio) were washed three times with PBS and subsequently centrifuged at 2000 × g, 5 min, and 4 °C. A 1:20 dilution of the red blood cells in PBS was incubated with various concentrations of the peptides in a 96‐well plate at 37 °C for 24 h. Untreated red blood cells were used as a negative control, and red blood cells treated with 3.3% Triton‐X100 as a positive control. After centrifugation at 1500 × g for 3 min, the supernatant was transferred into a new 96‐well plate and the absorption was measured at 540 nm using a Tecan infinite M200 plate reader.

##### Flow Cytometry Analysis

To quantify the cellular uptake of the peptides, flow cytometry measurements were performed. For this, 1.3 × 10^5^ HFF‐1 cells and 1.7 × 10^5^ BxPC‐3 cell were seeded in a 24‐well plate and grown to 80%–90% subconfluency. On the next day, cells were incubated with 10 μm CF‐labeled peptide in a serum‐free medium at 37 °C for 30 min. Untreated cells served as negative controls. Afterward, cells were washed with PBS and detached by 0.5 mg ml^−1^ trypsin‐EDTA lacking phenol red. Cells were resuspended in Dulbecco's Modified Eagle's Medium with high glucose levels, 10% FBS, and 4 mm glutamine, but without phenol red. The internalization was measured with a Guava easyCyte instrument (Merck). 10^4^ cells were analyzed per well using GRN‐B (525/30) laser.

##### Live Cell Imaging by Fluorescence Microscopy

For live cell fluorescence microscopy, 2.7 × 10^4^ HFF‐1 or 3.5 × 10^4^ BxPC‐3 and PANC‐1 cells were seeded in a μ‐slide eight‐well plate (ibidi) and grown to 70%–80% subconfluency. Cells were incubated with 10 μm of the CF‐labeled peptides at 37 °C for 30 min. For the last 10 min of incubation time, Hoechst 33 342 nuclear dye was added to stain the nuclei. After quenching background fluorescence by trypan blue (150 μm in 0.1 m acetate buffer, pH 4.1) for 10 s, cells were washed three times with PBS, and complete medium was added. Fluorescence was imaged using the Keyence BZ‐X810 with a 60x immersion oil objective and images were processed with Fiji.

For colocalization studies with the ER, 1.5 × 10^4^ PANC‐1 cells were seeded in a μ‐slide eight‐well plate (ibidi) and grown overnight. On the next day, CellLight‐red fluorescent protein (ER‐RFP), BacMam 2.0 reagent (ThermoFisher) was added and incubated for 48 h. Afterward, cells were treated with 10 μm CF‐labeled peptide at 37 °C for 30 min and the nuclei were stained with Hoechst 33 342 for the last 10 min. Cells were washed five times with PBS and complete medium was added. Finally, the fluorescence was imaged using an UltraView VoX spinning disk confocal microscope (Perkin Elmer, Waltham Massachusetts, USA) with a Plan‐Apo Tirf 60x/1,49 Oil DIC objective, and the resulting images were processed with Fiji.

For colocalization studies with lipid droplets, 3 × 10^4^ BxPC‐3 and PANC‐1 cells were seeded in a μ‐slide eight‐well plate (ibidi) and grown overnight at 37 °C. On the next day, cells were co‐incubated with a 1:1000 dilution of a distyryl Bodopy lipid droplet stain, which was synthesized and kindly provided by Engelhardt et al.^[^ ^53^ ^]^ and 10 μm of CF‐CaaX‐1 in serum‐free medium for 30 min at 37 °C. Cells were washed three times with serum‐free medium and finally kept in complete medium. Imaging was performed using an UltraView VoX spinning disk confocal microscope (Perkin Elmer, Waltham Massachusetts, USA) with a Plan‐Apo Tirf 60x/1,49 Oil DIC objective, and the resulting images were processed with Fiji.

For colocalization studies with the Golgi apparatus, 3 × 10^4^ BxPC‐3 and PANC‐1 cells were seeded in a μ‐slide eight‐well plate (ibidi) and grown overnight at 37 °C. Cells were incubated with a 1:100 dilution of BODIPY TR ceramide complexed to BSA (Thermo Scientific) in serum‐free medium for 30 min at 37 °C. Cells were then treated with 10 μm CF‐CaaX‐1 for 30 min and then with Hoechst 33 342 for 10 min at 37 °C. Cells were washed 4 times with serum‐free medium. Imaging was performed using an UltraView VoX spinning disk confocal microscope (Perkin Elmer, Waltham Massachusetts, USA) with a Plan‐Apo Tirf 60x/1,49 Oil DIC objective, and the resulting images were processed with Fiji.

##### Cell Lysates and Western Blotting

10^6^ BxPC‐3 or PANC‐1 cells were cultured in 6‐well plates to ≈90% subconfluency. For the experiment, in which the phosphorylation states and expression levels were investigated, cells were incubated with 30 μm CaaX‐1 in a serum‐free medium at 37 °C for 6 h or 24 h. Cells were washed twice with PBS and detached using trypsin‐EDTA. After resuspending cells in full medium, they were centrifuged at 1250 rpm and 4 °C for 5 min. Then, the supernatant was removed, and cells were resuspended in PBS and again centrifuged under the same conditions. The arising pellet was lysed in lysis buffer (25 mm Tris, 150 mm NaCl, 1 mm TCEP, 2 mm EDTA, 1% Triton‐X100, and pH 7.4) supplemented with Halt Protease and phosphatase inhibitor cocktail (Thermo Fisher) at 4 °C for 35 min under rotation. Alternatively, cells were washed two times with PBS, treated with lysis buffer and scraped with a cell scraper. The cell lysate was then rotated at 4 °C for 35 min. Cell lysates were centrifuged at 20 187 × g and 4 °C for 30 min to remove cell debris and Laemmli buffer was added to the supernatant. After denaturation at 95 °C for 5 min, proteins of the cell lysates were separated by Tricine‐SDS‐PAGE using a 6% or 10% polyacrylamide gel. Proteins were transferred from the gel onto a PVDF membrane by semi‐dry (100 mV, 1.5 h) or wet (350 mA, 2.5 h) blotting methods, and blocked in 5% milk powder in PBS‐T or 5% BSA in PBS‐T at room temperature for 1 h. Afterward, the membrane was incubated with the primary antibody at 4 °C overnight. After washing three times with PBS‐T, the membrane was incubated with the secondary antibody at room temperature for 1.5 h, and after further three washing steps with PBS‐T, the membrane was developed using SignalFire ECL Reagent (CST). To get a loading control, the membrane was washed twice in PBS and subsequently shaken two times in stripping buffer (0.2 m glycine 0.1% sodium dodecyl sulfate (SDS), 1% Tween 20, pH 2.2) at room temperature for 10 min. After further two washing steps with each of PBS and PBS‐T, the membrane was again blocked and incubated with an anti‐β‐actin antibody horseradish peroxidase (HRP) conjugate (Santa Cruz, sc‐47 778 HRP, 1:1000) at room temperature for 1.5 h. Development of the membrane was performed as described above.

The following primary antibodies were used: anti‐pAKT (CST Cat#4060, 1:1000); anti‐pMEK1/2 (CST Cat#9154, 1:1000); anti‐pERK1/2 (CST Cat#4370, 1:1000); anti‐NF1 (proteintech Cat#27 249‐1‐AP, 1:500); anti‐SOS1 (SCB Cat#sC‐17 793, 1:500); anti‐HRas (proteintech Cat#18 295‐1‐AP, 1:500); anti‐KRas (SCB Cat#sc‐517 599, 1:1000); anti‐caveolin‐1 (SCB Cat#sc‐53 564, 1:1000); anti‐GAPDH (SCB Cat#sc‐32 233, 1:1000) anti‐β‐actin HRP conjugate (SCB Cat#sc‐47 778 HRP, 1:1000). The following secondary antibodies were used: anti‐rabbit‐HRP conjugate (CST Cat#7074S, 1:1000); anti‐mouse‐HRP conjugate (CST Cat#7076S, 1:1000).

##### Membrane Fractionation

PANC‐1 cells were grown in complete medium in a 10 cm petri dish until ≈90% confluency. Cells were incubated with 30 μm CaaX‐1 in a serum‐free medium for 3 h at 37 °C. Cells incubated with serum‐free medium served as a negative control. Cells were washed with PBS, detached using trypsin‐EDTA, and resuspended in a complete medium. The cell suspension was centrifuged at 300 × g and 4 °C for 5 min. After removing the supernatant, cells were washed two times in PBS and the cell pellet was resuspended in membrane permeabilization buffer (10 mm piperazine‐N,N'‐bis(2‐ethanesulfonic acid (PIPES), 300 mm sucrose, 100 mm sodium chloride, 5 mm EDTA, 0.015% digitonin w/v, 1:100 diluted protease inhibitor cocktail, pH 6) as described by^[^ ^67^ ^]^ and incubated for 15 min on ice. Then, the cell suspension was centrifuged at 1000 × g and 4 °C for 10 min, the supernatant comprising the cytosolic fraction was transferred into a new reaction vessel and the pellet was resuspended in lysis buffer (10 mm PIPES, 300 mm sucrose, 100 mm sodium chloride, 5 mm EDTA, 0.2% triton X‐100 w/v PANC‐1 cells were grown in complete medium in a 10 cm petri dish until ≈90% confluency. Cells were incubated with 30 μm CaaX‐1 in serum‐free medium for 3 h at 37 °C. Cells incubated with serum‐free medium served as negative control. Cells were washed with PBS, detached using trypsin‐EDTA, and resuspended in complete medium. The cell suspension was centrifuged at 300 × g and 4 °C for 5 min. After removing the supernatant, cells were washed two times in PBS and the cell pellet was resuspended in membrane permeabilization buffer (10 mm PIPES, 300 mm sucrose, 100 mm sodium chloride, 5 mm EDTA, 0.015% digitonin w/v, 1:100 diluted protease inhibitor cocktail, pH 6) as described by^[^ ^67^ ^]^ and incubated for 15 min on ice. Then, the cell suspension was centrifuged at 1000 × g and 4 °C for 10 min, the supernatant comprising the cytosolic fraction was transferred into a new reaction vessel and the pellet was resuspended in lysis buffer (10 mm PIPES, 300 mm sucrose, 100 mm sodium chloride, 5 mm EDTA, 0.2% triton X‐100 w, 1:100 diluted protease inhibitor cocktail, pH 7.4) as described by R. Rampado et al.^[^ ^67^ ^]^ After 30 min incubation on ice, the suspension was centrifuged at 5000 × g and 4 °C for 15 min, and the supernatant, including the membrane protein fraction was transferred into a new reaction vessel. Both fractions were diluted in Laemmli buffer, heated at 95 °C for 10 min, and used for western blot analysis.

##### Immunofluorescence Staining

Heat sterilized coverslips were placed in a sterile 12‐well plate and coated with poly‐L‐lysine by incubating the coverslips with a poly‐L‐lysine solution for 1 h at 37 °C. Afterward, the coverslips were washed three times with PBS. 2.25 × 10^5^ PANC‐1 cells were seeded onto the coverslips and grown at 37 °C o/n. Cells were incubated with 30 μm CaaX‐1 in serum‐free medium at 37 °C for 3 h. After washing with PBS, fixation was performed by incubating cells with 4% paraformaldehyde at 20 °C for 15 min. Cells were washed with PBS and blocked with 3% bovine serum albumin (BSA) in PBS for 30 min. Then, cells were incubated with a KRas‐Alexa555 antibody conjugate (CST Cat#64 520, 1:100) in 0.1% Tween‐20% and 3% BSA in PBS at 4 °C o/n. Cells were washed with PBS and incubated with Hoechst 33 342 (1:1000) in PBS at 20 °C for 15 min. Cells were washed three times with PBS and subsequently mounted using ProLong diamond mounting medium (Thermo Scientific) and sealed with nail polish. Finally, cells were imaged using a Keyence BZ‐X810 with a 60x immersion oil objective and images were processed with Fiji.

## Conflict of Interest

The authors declare no conflict of interest.

## Supporting information

Supplementary Material

## Data Availability

The data that support the findings of this study are available from the corresponding author upon reasonable request.
