# Supplementary material for: Impact of Mutational Status on Intracellular Effects of Cell‐Permeable CaaX Peptides in Pancreatic Cancer Cells
Source: Chembiochem. 2025 Apr 24;26(10):e202401076. doi: 10.1002/cbic.202401076 (PMC12117442; doi:10.1002/cbic.202401076)
Supplement: Supplementary file 1 — Supplementary Material [file CBIC-26-e202401076-s001.pdf]

Supporting Information

Impact of Mutational Status on Intracellular Effects of Cell-Permeable CaaX Peptides in Pancreatic Cancer Cells

Merlin Klußmann[a], Martin Matijass[a], and Ines Neundorf*[a]

[a] Dr. Merlin Klußmann, Martin Matijass, Prof. Dr. Ines Neundorf
Department of Chemistry and Biochemistry, Institute of Biochemistry
University of Cologne
Zuelpicher Str. 47a, 50674 Cologne
E-mail: ines.neundorf@uni-koeln.de
Web address: https://neundorflab.uni-koeln.de

Table of contents

Peptides synthesized within this work **2**
LC-MS analysis of the synthesized unlabeled peptides **3**
LC-MS analysis of the synthesized CF-labeled peptides **4**

Table S1: Peptides used within this work.

Peptide	Sequence	MW $_{calc}$ [Da]	MW $_{exp}$ [Da]	Net charge	Purity [%]
CaaX-1	GLRKRLRKFRNK-SKTK-CVIM-OH	2463.08	2463.84	+9	>99
SaaX-1	GLRKRLRKFRNK-SKTK-SVIM-OH	2447.02	2447.76	+9	>99
CaaX-2	GLRKRLRKFRNK-GCMSCK-CVLS-OH	2584.22	2585.01	+8	>99
SaaX-2	GLRKRLRKFRNK-GCMSCK-SVLS-OH	2568.15	2568.96	+8	>97
CaaX-3	GLRKRLRKFRNK-GCMSCK-CVGS-OH	2528.11	2528.84	+8	>97
sC18*	GLRKRLRKFRNK-NH_2	1571.92	1571.45	+8	>98
CF-CaaX-1	CF-GLRKRLRKFRNK-SKTK-CVIM-OH	2821.40	2822.44	+8	>98
CF-SaaX-1	CF-GLRKRLRKFRNK-SKTK-SVIM-OH	2805.34	2806.21	+8	>98
CF-CaaX-2	CF-GLRKRLRKFRNK-GCMSCK-CVLS-OH	2942.54	2943.37	+7	>99
CF-SaaX-2	CF-GLRKRLRKFRNK-GCMSCK-SVLS-OH	2926.47	2927.54	+7	>98
CF-CaaX-3	CF-GLRKRLRKFRNK-GCMSCK-CVGS-OH	2886.43	2887.17	+7	>98
CF-sC18*	CF-GLRKRLRKFRNK-NH_2	1929.26	1929.76	+7	>99

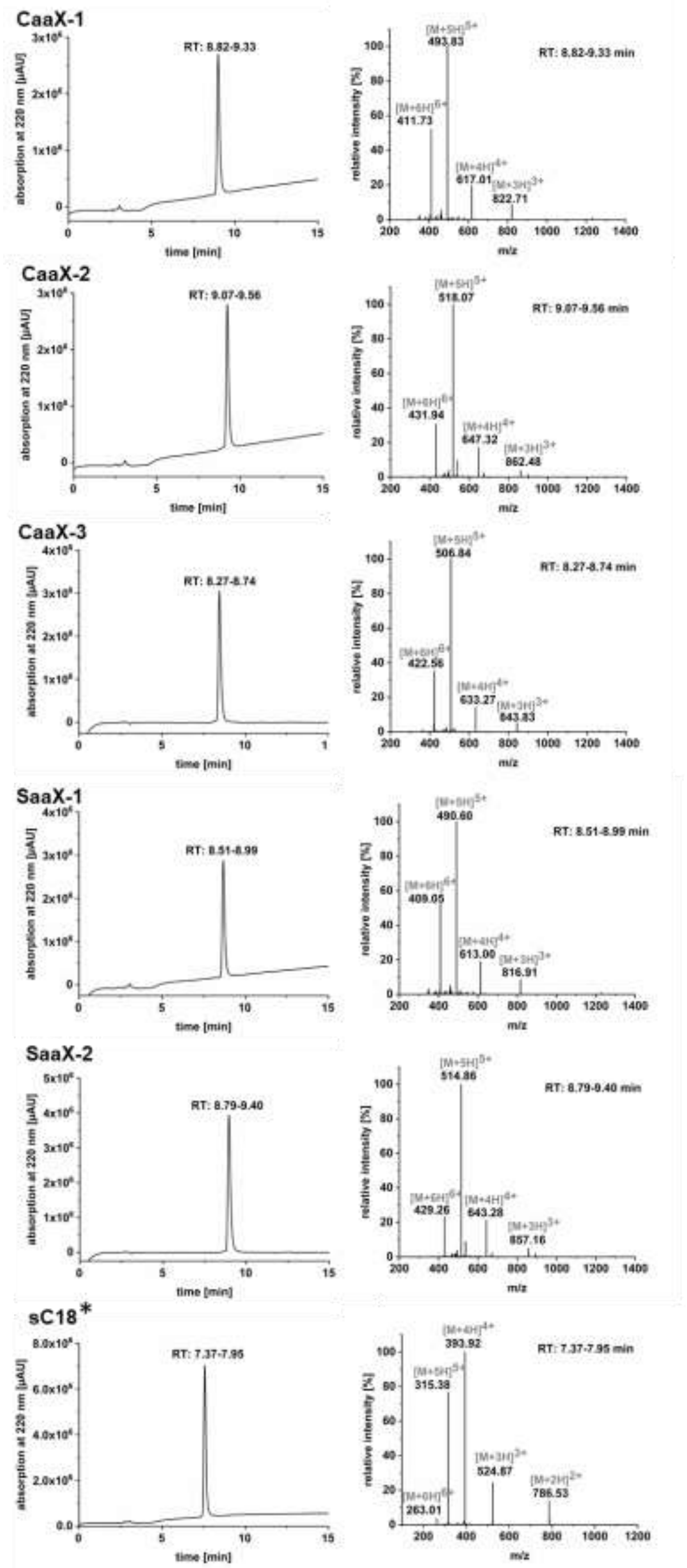

Figure S1: LC-MS analysis of cell-permeable CaaX- and control peptides using a gradient of 10-60 % ACN in water and 0.1 % TFA in 15 min.

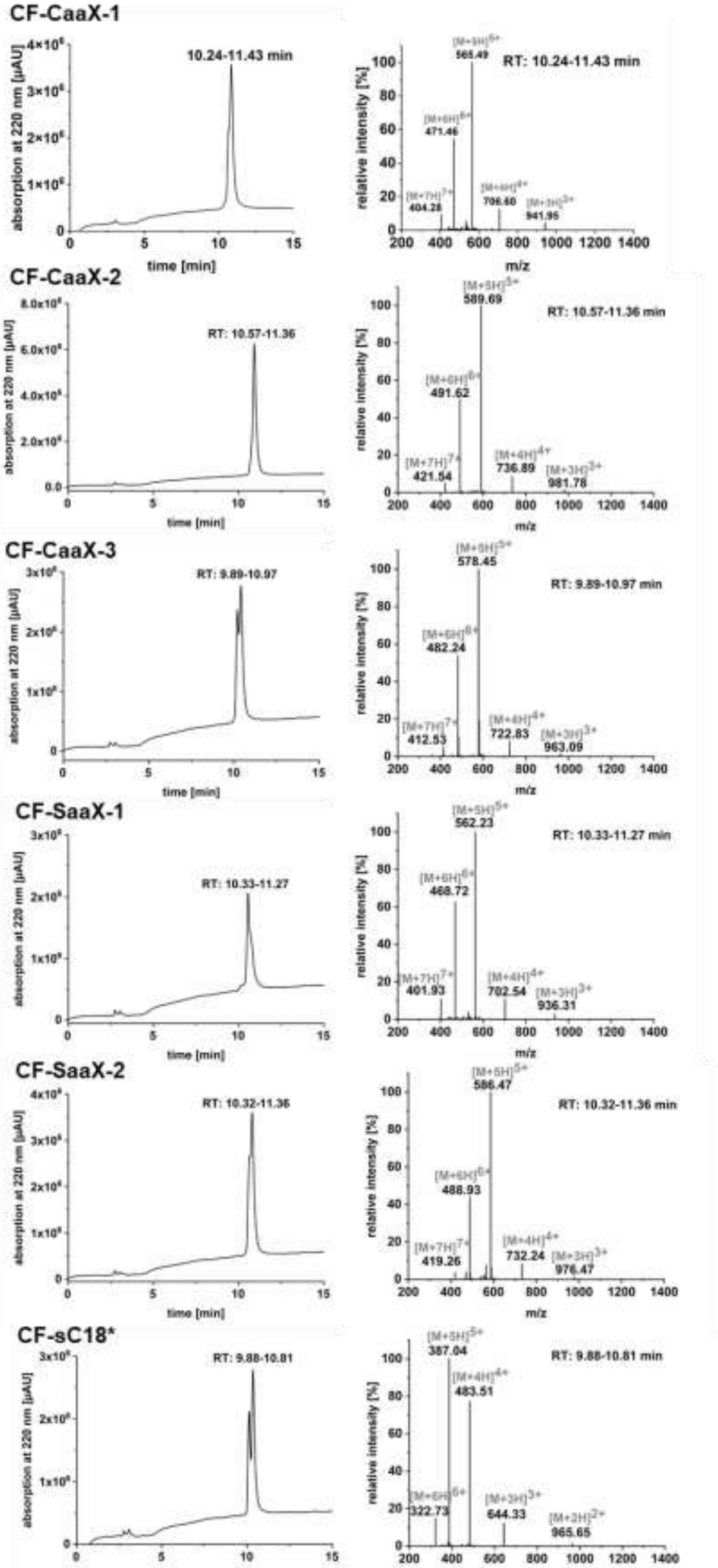

Figure S2: LC-MS analysis of CF-labeled cell-permeable CaaX- and control peptides using a gradient of 10-60 % ACN in water and 0.1 % TFA in 15 min.
